# Antimicrobial drug use and its association with antimicrobial resistance in fecal commensals from cows on California dairies

**DOI:** 10.3389/fvets.2024.1504640

**Published:** 2025-02-10

**Authors:** Essam M. Abdelfattah, Pius S. Ekong, Emmanuel Okello, Tapakorn Chamchoy, Betsy M. Karle, Randi A. Black, Wagdy ElAshmawy, David Sheedy, Deniece R. Williams, Terry W. Lehenbauer, Barbara A. Byrne, Sharif S. Aly

**Affiliations:** ^1^Veterinary Medicine Teaching and Research Center, School of Veterinary Medicine, University of California, Davis, Tulare, CA, United States; ^2^Department of Animal Hygiene and Veterinary Management, Faculty of Veterinary Medicine, Benha University, Al Qalyubiyah, Egypt; ^3^Department of Population Health and Reproduction, School of Veterinary Medicine, University of California, Davis, Davis, CA, United States; ^4^Cooperative Extension, Division of Agriculture and Natural Resources, University of California, Orland, CA, United States; ^5^Cooperative Extension, Division of Agriculture and Natural Resources, University of California, Santa Rosa, CA, United States; ^6^Department of Internal Medicine and Infectious Diseases, Faculty of Veterinary Medicine, Cairo University, Giza, Egypt; ^7^Department of Pathology, Microbiology, and Immunology, School of Veterinary Medicine, University of California, Davis, Davis, CA, United States

**Keywords:** antimicrobial resistance, antimicrobial drug use, antibiotics, dairy cows, California, season, region, dose

## Abstract

The current study objective was to investigate the risk factors associated with the isolation of antimicrobial-resistant *Escherichia coli*, *Enterococcus* spp., and *Streptococcus* spp. (ES) from the feces of dairy cows in California (CA). A longitudinal study was conducted on ten dairies, and a random sample of cattle (late pregnant heifers and dry cows) stratified by each herd’s parity distribution were followed monthly from close-up to 120 days in milk during fall to winter 2018 (winter season) and spring to summer 2019 (summer season). Gastrointestinal commensals were isolated from fecal samples and tested for antimicrobial susceptibility using the broth microdilution method against a selected panel of antimicrobial drugs (AMD). Eight dairies used blanket AMD therapy at dry-off for all lactating cows, while the remaining two dairies did not use any AMD treatment at dry-off. Clinical mastitis was identified as the most common indication for AMD use across the study dairies. Intramuscular administration of ceftiofur hydrochloride to treat lameness and unknown disease during lactation was significantly associated with the isolation of tetracycline-resistant fecal *E. coli*. Resistance to ceftiofur, tetracycline, or trimethoprim-sulfamethoxazole in fecal *E. coli* was significantly higher in the winter than in the summer season. In contrast, resistance to tetracycline, florfenicol, tilmicosin, tildipirosin, or tiamulin in fecal gram-positive commensals was significantly higher in the summer than in the winter. In conclusion, AMD usage practices and seasonal variations significantly influenced the AMR of *E. coli* and ES in the feces of dairy cattle.

## Introduction

Antimicrobial resistance (AMR) is a significant global health issue with considerable financial and health implications for both humans and animals (1). The use of antimicrobial drugs (AMD) and the subsequent development of AMR involves a complex, multifactorial process (1). In response to societal concerns about AMR in livestock, the US Food and Drug Administration (FDA) implemented the final rule of the Veterinary Feed Directive (VFD) in 2017, along with additional guidance. These regulations aim to ensure the judicious use of all medically important antimicrobial drugs (MIADs) administered through feed or water to food-producing animals (2). In 2018, California (CA) implemented Senate Bill 27 (SB 27), requiring veterinary prescriptions under a valid veterinarian-client-patient relationship (VCPR) for all other dosage forms of MIADs used for livestock and administered by routes other than in feed or water, which includes injectable, intramammary (IMM), and other oral AMD dosage forms (3). The CA state law has resulted in moving the AMDs that were available in livestock supply and feed stores as over-the-counter (OTC) products to prescription only under a valid VCPR. Similarly, the FDA issued Guidance for Industry #263 in 2019, moving OTC AMD nationwide to prescription-only status for all animal uses, and in 2021, initiated a two-year timeline for full implementation beginning on 11th June 11, 2023 (4).

Fecal commensals, including *Escherichia coli* and *Enterococcus* spp., have been used as indicator organisms in various studies on AMR because they can acquire resistance genes and act as a reservoir for the spread of resistance genes (5). Commensal bacteria in livestock can serve as a reservoir for resistance genes that could be transferred to other bacteria that may cause cattle disease (6). To control the spread and prevent AMR in commensal bacteria, it is important to identify the risk factors that are associated with such resistance. Understanding the association between the use of AMD and AMR in animal production systems will help develop effective control measures (7). Differences in exposure to AMD, management practices, and exposure to other risk factors, such as the age of the animal, herd size, sampling season, and region, were found to be associated with the development of AMR (7, 8). Previous studies have focused on AMR in calves (9, 10), AMR bacteria isolated directly from clinical mastitis cases (11, 12), or focused on AMR dynamics related to a specific drug (8). However, there is little information regarding the effect of locally applied IMM AMDs or systemically administered AMDs on Gram-negative or Gram-positive enteric commensals in dairy cows.

To the best of our knowledge, this is the first study conducted on California dairies after the implementation of SB 27 to study the effect of AMD use on AMR of fecal commensals. The current study uniquely followed cow cohorts across various regions and seasons, providing comprehensive insights into the regional and seasonal dynamics of AMR. The primary objective of this longitudinal study was to explore the associations between herd demographics, management practices, and AMD use as predictors of AMR in fecal commensal bacteria isolated from cows on CA dairies followed from the dry period to 120 days in milk (DIM) post-calving. We hypothesized that various factors contribute to the development of AMR in fecal commensal bacteria isolated from dairy cows. These factors include herd demographics such as herd size, breed, region, and season; management practices including dry-off protocols and disease condition; and the use of antimicrobial drugs, specifically the timing of AMD administration and the type of AMDs used, whether systemic or IMM.

## Materials and methods

### Study herds and sample collection

The study was approved by the University of California Davis’ Institutional Animal Care and Use Committee (protocol number 19871). The current study was part of a prospective longitudinal study conducted to describe the epidemiology and patterns of AMR phenotypes among fecal commensal bacteria from adult dairy cows in CA dairies across the state’s regions and seasons. Details of the study herds, management practices, sampling, and laboratory procedures were described in (13). The current study was conducted on 10 dairies, with each dairy enrolling two cohorts of cows over two distinct seasons. The first cohort was sampled during the fall and winter of 2018 and is referred to as the winter cohort. The second cohort was sampled during the spring and summer of 2019 and is referred to as the summer cohort. Each dairy was visited five times per cohort at intervals of 4–5 weeks. A random sample of 12 cows per dairy per cohort was enrolled before calving (close-up stage), and up to 120, with a total of 240 cows, were enrolled in this study. During the 12-month study period, a total of 240 cows were identified and enrolled using a parity-stratified random sample of the 10 study herds (12 cows per dairy over two seasons). The study dairies were distributed throughout CA’s three dairy regions: three in Northern California (NCA), two in Northern San Joaquin Valley (NSJV), and five in Greater Southern California (GSCA) based on Love et al. (14).

In each season, fecal samples were collected during monthly sampling points from a stratified random sample of 12 late-pregnancy heifers and cows, identified proportional to each herd’s parity distribution, from close-up (approximately 2 weeks prior to calving) to 120 DIM. Therefore, the number of pregnant heifers enrolled corresponded to the frequency of the first lactation cows in each study herd. Upon calving, these heifers were sampled as cows up to 120 DIM (DIM). Fecal samples were collected from enrolled cows monthly from 2 weeks before calving up to 120 DIM with a total of five sampling points (closeup, 30, 60, 90, and 120 DIM). Fecal samples were collected in 50 mL polypropylene tubes and transported to the laboratory on wet ice for culture within 24 h. Data on the study cows’ body condition and fecal scores were also collected during sampling. Body condition score (BCS) was assessed on a 5-point scale [1 = thin, 3 = average, and 5 = obese, according to (15)]. Each cow’s fecal consistency was assessed on a 3-point scale (1 = normal, 2 = loose, 3 = watery), which had been used previously in cattle (16).

The collected fresh fecal samples were directly plated onto *E. coli* ChromoSelect agar and Enterococci ChromoSelect agar (Sigma–Aldrich, St. Louis, MO, USA) for isolation of *E. coli* and *Enterococcus* spp., respectively, within 24 h of fecal sample collection. Due to a recent taxonomical update that distinguishes the genus *Enterococcus faecalis* and other members of the genus from *Streptococcus* spp., Enterococci ChromoSelect agar was only able to identify *Enterococcus* spp. and *Streptococcus* spp. by colony appearance (17). Therefore, colonies isolated from the Enterococci ChromoSelect agar from here onward were referred to as *Enterococcus* spp./*Streptococcus* spp. (ES). From each fecal sample, two isolated *E. coli* and two ES colonies were selected from ChromoSelect agars for antimicrobial susceptibility testing using the broth microdilution method (18) using the Sensititre^™^ Bovine BOPO7F Plate (Thermo Scientific, Remel Inc., Lenexa, KS, USA). Antimicrobial susceptibility testing was performed against the panel of AMD, including ampicillin, penicillin, ceftiofur, danofloxacin, enrofloxacin, florfenicol, gamithromycin, gentamicin, neomycin, sulfadimethoxine, spectinomycin, trimethoprimsulfamethoxazole, tetracycline, tiamulin, tilmicosin, tildipirosin, tulathromycin, and tylosin tartrate. Subsequently, isolates were classified as susceptible or resistant (intermediate isolates were classified as resistant) based on MIC breakpoints set by the Clinical and Laboratory Standards Institute if available (19); otherwise, MIC breakpoints were suggested by other publications as detailed in (13). Susceptibility testing quality control measures were conducted weekly using five control strains, including *E. coli* ATCC 35218, *E. coli* ATCC 25922, *Enterococcus faecalis* ATCC 29212, Strep. pneumoniae ATCC 49619, and *Histophilus somni* 700025.

Data on additional predictors were collected at the start and end of each sampling season. Specifically, each dairy’s enrollment questionnaire was completed to characterize its herd management practices and AMD use for dry cow therapy (DCT) and other therapeutic uses. The questionnaire was also completed for each dairy after the sampling periods, focusing on documenting changes in AMD use or management practices that the dairy management adopted during the study period. The questionnaire was reimplemented from a previously published antimicrobial stewardship survey about herd demographics, health management and antibiotic use, and antimicrobial stewardship practices and perspectives (20).

### Antimicrobial drug use data

Data regarding the AMD exposure of the 12 individual study cows at each dairy during each season were systematically recorded in eight of the 10 study herds using computer management systems—Dairy Comp 305 for five herds and DHI Plus for three herds. Backup copies of these records were collected at each sampling visit across the two study seasons, totaling 10 backups per dairy. The remaining two study dairies maintained their records on paper, which were accessed monthly during each sampling visit. The individual cow data included the animal identification number, the AMD used, dose, route of administration, and date of AMD administration in addition to DCT. In addition, treatment protocols for each dairy were accessed through their respective record-keeping software. Treatment data and antimicrobial resistance data were time-matched in a relational database (Microsoft Access, Microsoft Corp., Redmond, WA, USA), preserving the temporality of the study cows’ AMD exposure with respect to their fecal sample isolates’ AMR phenotype.

Adapted from (21, 22), the treatment records were stratified by use categories (UC) into a dry cow, clinical mastitis, lameness, metritis, gastrointestinal, and unknown disease condition or syndrome. The UC dry cow represented IMM administration of long-acting AMD treatments administered at the end of lactation, while the UC clinical mastitis represented IMM AMD treatments to treat clinical mastitis during the lactation. The UC lameness represented the systemic administration of AMD for treatment of lameness or foot rot as detected by lameness signs and/or hoof trimmer examination. The UC gastrointestinal represented the systemic AMD for the treatment of diarrhea and other disease conditions related to the gastrointestinal system. The UC unknown disease represented the AMD administered to sick animals without identifying the specific cause (cow record information did not specify the underlying disease condition). Multiple measures were calculated to standardize the AMD use across the study dairies based on (22). These measures included the concentration of active substance (AS) in each AMD product. The active substance concentration was identified from the product label and, where needed, converted to milligrams (mg/mL).

Products containing active substances (AS) indicated in International Units (IU) instead of milligrams (mg), such as procaine penicillin, were converted to mg using a conversion factor of 1,000 IU per mg (23). For each AS in our study, the following measures were calculated based on the formulas used in (22): gram per administration (g/admin = grams of AS per administration); gram per regimen (g/reg = grams of AS per regimen), where a regimen refers to the prescribed course of treatments; and administration per regimen (Admin = number of administrations per regimen) (21).

In addition, the Defined Daily Dose for the study (DDDstudy) was calculated for cows randomly selected from their respective herds. This estimation aimed to characterize the typical dose that a standard dairy cow (680 kg) would receive if treated according to the FDA-approved label dosage. The dosage was calculated in units of mg/kg/day. A single administration was a drug product administered at a single cow treatment administration event. Multiple administrations were considered a single regimen when product administrations were consecutive, with no time gap between administrations greater than an interval of 2 days. Treatment intervals were explored, and a new case of the same disease was identified if a gap of 5 or more days was observed between treatments for the same cow.

### Sample size calculations

The number of herds included in the study was determined using a convenience sample of 10 dairies. These dairies were selected to represent different milk sheds across California, ensuring geographic diversity and variability in management practices. Additionally, this number was chosen to facilitate the practical management of data collection across the study period.

The study sample size was determined *a priori* using the formula for comparing a dichotomous outcome between two groups with repeated measures, as described in Equation 1 by Brown and Prescot (24). The number of dairy cows to enroll in a given herd was based on the difference (∆) in the proportion of cows with resistant fecal commensals in AMD-treated cows (Group 1, *P*_1_ = 33%) compared to untreated cows (Group 2, *P*_2_ = 3%), where ∆ is measured on the logit scale such that ∆ = logit(P_1_) – logit(P_2_) for *m* repeated measures (5 sampling events/cow) assuming a compound symmetry covariance structure with a correlation *ρ* (where *ρ* = 0.25), and the quantity *ν,* the inverse of the P(1-P), where P is the mean of group proportions P_1_ and P_2_.


(1) $$n=2{\left(z_{1-\alpha /2}+z_{\beta }\;\right)}^{2}v\frac{\left[1+\left(m-1\right)\rho \right]}{m\Delta^{2}}$$

A two-sided hypothesis test framework tested a null hypothesis that the OR = 1 and an alternative hypothesis that OR ≠ 1 with an alpha of 0.05 and a power of 80%.

Given these inputs, a sample size of 12 cows (treated and untreated) per herd was deemed necessary to detect a significant difference in the proportions of resistant fecal commensals in AMD-treated and untreated cows. Given that 10 herds were enrolled and followed up over 2 seasons, a total of 240 cows were enrolled (12 cows/herd x 2 seasons x 10 herds = 240).

### Statistical analyses

Descriptive statistics were used to calculate the proportion of AMR at the isolate and cow levels. Resistance status at the cow level was defined as one or more isolates resistant to one or more AMD at one or more time points during the collection time from calving up to 120 DIM. Cow-level AMR incidence was estimated for each specific drug and for *E. coli* and ES separately, as the total number of cows with at least 1 AMR isolate was divided by the total number of cows sampled. The mean, median, standard deviation, minimum, and maximum were calculated for AMD use measures, including the g/admin, g/reg, Admin, and DDD.

Collinearity of all potential explanatory variables was checked using Spearman’s rank correlation statistic. Mixed effects logistic regression models were specified for AMR outcomes at the animal level. The random effect structure included the cow as a random intercept and the sampling point as a random slope to account for the lack of independence of observations over time within the same cow. Robust standard error estimates were calculated with a clustered sandwich variance estimator that allowed for intragroup (farm-level) correlations. All independent variables, including AMD use, herd demographics (sampling region, sampling season, sampling points, herd average milk production, breed, milking herd average size, milking herd average somatic cell count (SCC), dry-off protocol), and animal-level factors (BCS, fecal score, disease condition, parity), were explored using univariate models for cow-level resistance to each AMD. Predictors associated with an outcome of interest at *p* ≤ 0.20 were considered for further modeling. A manual forward model-building approach was used, and variables were retained in the model if significant at *p* ≤ 0.05 or an improvement in model goodness of fit was observed using the Akaike Information Criterion (AIC), where a lower AIC estimate was considered a better fit. Confounding was assessed using the method of change in estimates, where a change of 30% or more in model estimates was considered evidence of confounding. All biologically meaningful interaction terms were explored using significance testing. Finally, previously excluded variables were offered once again to the resulting model and retained at *p* ≤ 0.05. The coefficients, odds ratios (OR), and their associated 95% confidence intervals were estimated in the final model for significant factors (*p* ≤ 0.05). All statistical analyses were performed using Stata 15 and 16 (Stata Corp, College Station, TX, USA).

## Results

### Descriptive statistics

The study herds included five herds in GSCA, two in NSJV, and three in NCA. The average number of milking cows in the study herds was 1,605 cows/herd, with a minimum of 130 cows/herd and a maximum of 5,000 cows/herd. The annual rolling herd average milk production was 11,390 ± 530.57 kg/cow/year. Regarding breed distribution, six herds were 100% Holstein, one herd was 100% Jersey, one was 100% crossbred, and two were mixed breeds. The herd average SCC was 140,000 ± 17,950 cells per mL.

The body condition score (BCS) of the enrolled cows, expressed as mean ± standard error (SE), was recorded on different days in milk (DIM). The body condition score of enrolled cows (mean ± SE) was 3.4 ± 0.01, 3.2 ± 0.02, 3.1 ± 0.02, 3.1 ± 0.02, 3.1 ± 0.02 during close-up, 30, 60, 90, 120 DIM respectively.

### Cow and isolate-level AMR

Culture of some fecal samples did not yield *E. coli* (68 samples) or ES (75 samples) after three culture attempts. Hence, a total of 2,169 *E. coli* and 2,157 ES isolates were available for antimicrobial susceptibility testing. Table 1 summarizes the proportion of *E. coli* and ES isolates with AMR against drugs that these species are otherwise known to be susceptible to. At the isolate level, the highest proportion of resistant *E. coli* was against florfenicol (83.31%), followed by sulfadimethoxine (32.45%) and tetracycline (16.82%). For ES, the highest proportion of resistant isolates was to tildipirosin (50.2%), followed by tilmicosin (47.90%), florfenicol (46.50%), and tiamulin (42.4%) (Table 1). Overall, cow-level incidence of AMR in *E. coli* ranged from 2.50 to 99.58%, while in ES, AMR ranged from 1.68 to 95.38% (Table 1).

**Table 1:** The proportion of *E. coli* and *Enterococcus/Streptococcus* spp. resistant to antimicrobial drugs and cow-level incidence of antimicrobial resistance based on fecal commensal resistance.

Antimicrobial resistance	Isolates proportion (%, *n*)	Cow-level incidence^1^		
		Winter cohort (%, *n*)	Summer cohort (%, *n*)	Overall (%, *n*)
*Escherichia coli*				
Ampicillin	1.10% (23)	10.83% (13)	9.16% (11)	10% (24)
Ceftiofur	1.93% (40)	20% (24)	5% (6)	12.50% (30)
Danofloxacin	4.01% (87)	39.16% (47)	9.16% (11)	24.16% (58)
Enrofloxacin	3.31% (72)	34.16% (41)	6.66% (8)	20.41% (49)
Gentamicin	0.32% (7)	5% (6)	0.00%	2.5% (6)
Neomycin	1.61% (35)	15.83% (19)	10% (12)	12.92% (31)
Spectinomycin	5.07% (110)	44.17% (53)	13.33% (16)	28.75% (69)
Florfenicol	83.31% (1,807)	99.16% (119)	100% (120)	99.58% (239)
Tetracycline	16.82% (365)	81.66% (98)	58.33% (70)	70% (168)
Sulfadimethoxine	32.45% (704)	90.83% (109)	86.66% (104)	88.75% (213)
Trimethoprim-sulfa	4.47% (97)	41.66% (50)	10% (12)	25.83% (62)
*Enterococcus/Streptococcus* spp.				
Penicillin	0.18% (4)	2.54% (3)	0.83% (1)	1.68% (4)
Ampicillin	0.23% (5)	11.01% (13)	9.20% (11)	10.10% (24)
Florfenicol	46.55% (1,004)	92.37% (109)	98.33% (118)	95.38% (227)
Tetracycline	15.25% (329)	55.10% (65)	64.20% (77)	59.66% (142)
Gamithromycin	11.54% (249)	47.46% (56)	62.50% (75)	55.04% (131)
Tildipirosin	50.19 (1,082)	83.90% (99)	97.50 (117)	90.76% (216)
Tilmicosin	47.91% (1,033)	83.05% (98)	98.33% (118)	90.76% (216)
Tulathromycin	7.64% (165)	36.44% (43)	48.33% (58)	42.44% (101)
Tylosin	3.19% (69)	23.73% (28)	17.50% (21)	20.59% (49)
Tiamulin	42.37% (914)	76.27% (90)	94.17% (113)	85.29% (203)

Cow-level antimicrobial resistance status was specified for each of the study’s 240 cows across both seasons except for two cows in the winter cohort from which *Enterococcus*/*Streptococcus* spp. could not be isolated.

1 Proportion estimate based on the respective bacteria isolated from at least one fecal sample during either season testing positive for antimicrobial drug resistance.

### Antimicrobial drug use

Eight of our study herds reported the use of blanket dry-cow therapy (BDCT) at dry-off, employing either IMM AMD alone or in combination with an internal teat sealant. The remaining two herds did not administer IMM AMD or teat sealant at dry-off.

**Figure 1:**
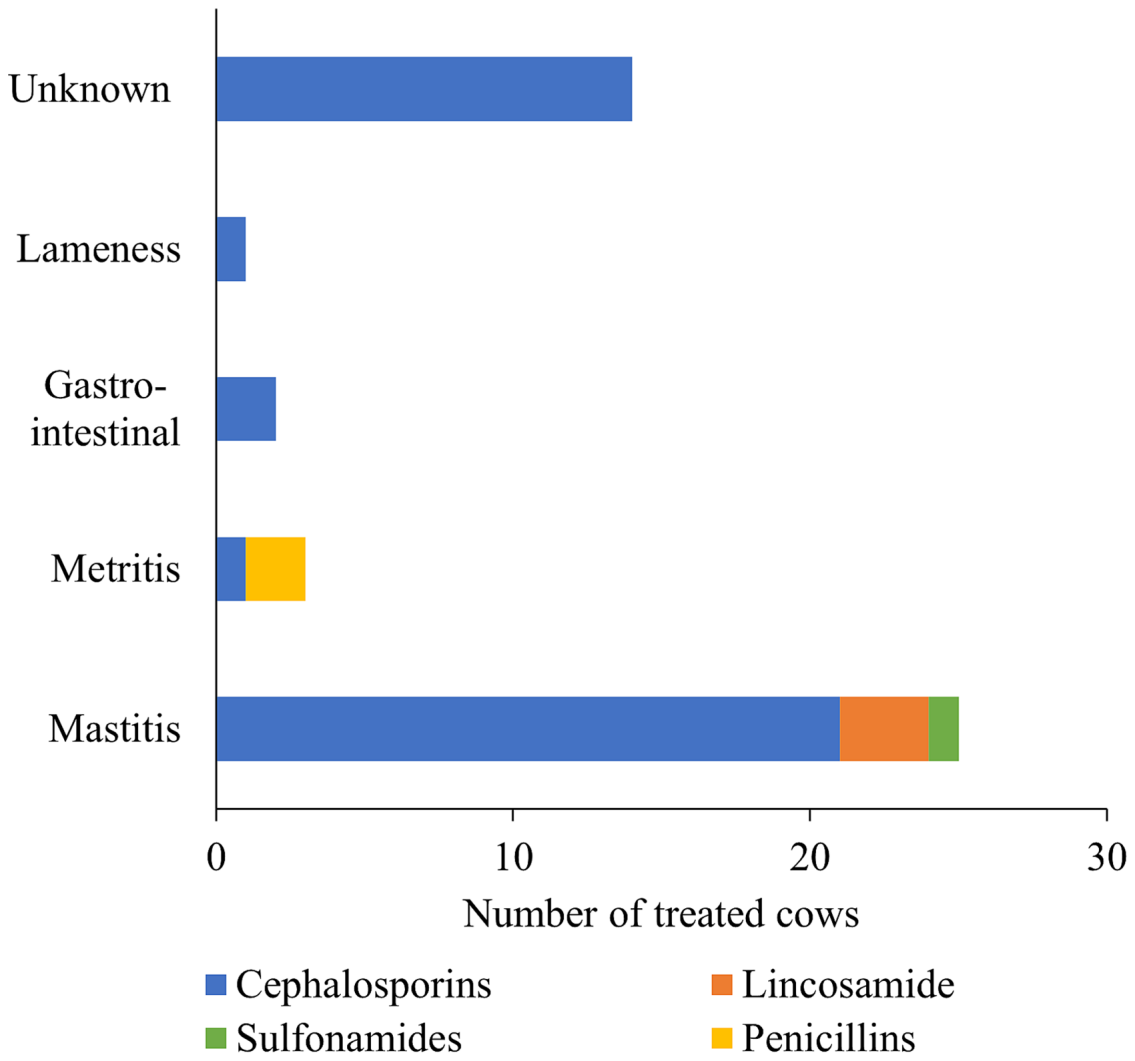
A stacked bar chart depicts the breakdown of therapeutic antimicrobial drugs (AMD) administered by drug class and the number of cows treated for different disease conditions. The AMD use information was collected for a random sample of 12 cows stratified by parity distribution on each of 10 California dairies and followed up from close up (approximately 2 weeks prior to calving) to 120 days in milk over two seasons (Winter and Summer).

For BDCT, four herds reported the use of ceftiofur hydrochloride at a dose of 2 g/regimen, three herds used cephapirin benzathine at a dose of 1.2 g/regimen, and one herd reported using a combination product containing procaine penicillin G (4 g/regimen) and dihydrostreptomycin (4 g/regimen). However, among 4 of the 12 cows enrolled during the summer season, each had only two functional quarters, resulting in a mean dose of 3.38 g/regimen for the combined AMD. In Supplementary materials, descriptive statistics of AMD administered to the study cows and drug mass per regimen are summarized in Supplementary Tables 1, 2. Of the 240 study cows, only 37 (15.4% ± 2.33) in the study dairies received AMD for treating clinical disease conditions. Specifically, during both study seasons, 41 cases among the 37 treated cows consisted of either clinical mastitis (*n* = 21), metritis (*n* = 3), gastrointestinal (*n* = 2), lameness (*n* = 1), or an unknown disease condition (*n* = 14) (Figure 1). The number of grams per administration for the active drug substances administered in our study is summarized in Supplementary Table 3.

Ceftiofur hydrochloride was administered as follows: an average rate of 2 g/administration intramammary for DCT, 1 g/admin intramuscular for the treatment of lameness, 0.75 g/admin subcutaneously for the treatment of clinical metritis, and 0.125 g/administration intramammary for the treatment of clinical mastitis.

Clinical mastitis cases were treated intramammarily with 0.125 g of ceftiofur hydrochloride per quarter. The overall ceftiofur hydrochloride regimen ranged from 0.25 to 1g/regimen, with a mean of 0.57 g/regimen across 2 to 8 administrations (Supplementary Tables 2–4).

One clinical mastitis case was treated with 0.125 g of ceftiofur hydrochloride per quarter administered intramammarily at 12-h intervals (Supplementary Table 1). Each administration, defined as treatment following a single restraint, counted as a separate event for a total of 2 administrations in a 24 hour period. Hence, despite such a cow being treated with a daily dose of 0.250 g ceftiofur hydrochloride, it had 0.125 g/administration. Cephapirin sodium was used for the treatment of clinical mastitis at a dose range from 0.2 to 0.4 g/reg, with a mean dose of 0.20 g/administration (adjusting for treatment of multiple simultaneous quarters with clinical mastitis), with a range of 1 to 2 administrations per regimen. Pirlimycin hydrochloride was used for the treatment of bovine clinical mastitis at a dose range from 0.15 to 0.2 g/reg, a mean dose of 0.05 g/administration, with a range of 3 to 4 administrations per regimen, and one dairy reporting a case with all 4 quarters treated for clinical mastitis for 4 days using pirlimycin. Records for one enrolled herd showed the use of sulfadimethoxine for the treatment of clinical mastitis at a dose of 100 g/reg, with a mean dose of 20 g/administration for a total of five administrations. Ceftiofur hydrochloride was used to treat lameness at 1 g per administration, with five administrations per regimen reported by study dairies. Cows diagnosed with clinical metritis were treated with ampicillin at a dose of 18.75 g/reg administered intramuscularly or ceftiofur hydrochloride at a dose of 2.25 g/reg administered subcutaneously. The DDD study for each active substance for treated cows is reported in Table 2. The mean calculated DDD study for ceftiofur hydrochloride for DCT was 2.94 mg/kg/day based on all four quarters receiving intramammary infusions at dry-off, while the average calculated DDDstudy for ceftiofur hydrochloride for treatment of clinical mastitis was 0.183 mg/kg/day after adjusting for clinical mastitis in multiple quarters in the same cow.

**Table 2:** Descriptive statistics of defined daily doses (DDDstudy) for each active substance administered on 8 California dairies to a random sample of 12 cows stratified by parity distribution on each of 10 premises followed up from close-up (approximately 2 weeks prior to calving) to 120 days in milk over two seasons (Winter and Summer) for a total of 240 cows.

Use category	Active substance	Number of Herds	Mean DDDstudy^1^	Median DDDstudy	Standard deviation DDDstudy	Min DDDstudy	Max DDDstudy	Product label dosage
Dry cow^2^^,^^3^	Ceftiofur hydrochloride (Intramammary)	4	2.941	2.941	0	2.941	2.941	500 mg/syringe/quarter
	Cephapirin benzathine (Intramammary)	3	1.764	1.794	0	1.764	1.764	300 mg/syringe/quarter
	Procaine Penicillin G/dihydrostreptomycin sulfate combination (Intramammary)	1	5.392	5.882	1.096	2.941	5.882	1,000 mg/quarter
Clinical mastitis	Ceftiofur crystalline free acid (Subcutaneous)^4^	1	4.41	4.41	–	4.41	4.41	6.6 mg/kg/72 h
	Ceftiofur hydrochloride (Intramammary)	6	0.183	0.183	0	0.183	0.183^5^	125 mg/syringe/quarter
	Cephapirin sodium (Intramammary)	2	0.294	0.294	0	0.294	0.294	200 mg/syringe/quarter
	Pirlimycin hydrochloride (Intramammary)	2	0.07	0.07	0	0.07	0.07	50 mg/syringe/quarter
	Sulfadimethoxine (Intravenous)	1	29.41	29.41	–	29.41	29.41	55 mg/kg/day (day 1) 27.5 mg/kg/day (>day 1)
Lameness	Ceftiofur hydrochloride (Intramuscular)	1	1.47	1.47	–	1.47	1.47	1.1–2.2 mg/kg/day
Metritis	Ampicillin (Intramuscular)	1	9.19	9.19	0	9.19	9.19	11.11 mg/kg/day
	Ceftiofur hydrochloride (Subcutaneous)	1	1.10	1.10	–	1.10	1.10	2.2 mg/kg/day
Gastro-intestinal	Ceftiofur hydrochloride (Subcutaneous)	1	1.29	1.29	0.26	1.10	1.47	1.1–2.2 mg/kg/day
Unknown^6^	Ceftiofur crystalline free acid (Subcutaneous)	1	4.41	4.41	–	4.41	4.41	6.6 mg/kg/72 h
	Ceftiofur hydrochloride (Intramuscular)	1	1.10	1.10	0	1.10	1.10	1–2.2 mg/kg/day

1 DDDstudy is a technical unit defined as the assumed average active substance per kg animal per day (mg/kg/day), assuming a cow weight of 680 kg. DDDstudy accounts for the use of long-acting drugs by incorporating the correction factor adjusted days of therapy, which in turn adjust for the longer time frame of therapy.

2 Two of the 10 dairies did not administer dry cow therapy at dry-off, and their enrolled study cows received no AMD during the study period.

3 The DDDstudy for intramammary products was assigned based on European Medicines Agency guidelines (2015), with four intramammary infusions (one per quarter) constituting one DDDstudy for dry-cow therapy.

4 Long-acting drug with time frame adjustment is needed to reflect the days the antibiotic is active. In the case of Ceftiofur, crystalline-free acid is administered up to two times, with the final second dose after 72 h of the first dose. Thus, a correction factor of 3 was applied to account for the extended activity period of the drug.

5 Reported at a quarter level for clinical mastitis to account for inflammation affecting more than 1 quarter. A lack of adjusting for multiple quarter infections simultaneously would falsely inflate the DDDstudy.

6 Cows received antibiotic treatment without a reported disease condition or specified cause.

### Generalized linear mixed models

Generalized linear mixed models with a logit link were specified for the following *E. coli* AMR outcomes: ceftiofur, tetracyclines, florfenicol, sulfadimethoxine, and trimethoprim-sulfamethoxazole. In addition, we could not specify *E. coli* models for resistance against gentamicin, spectinomycin, ampicillin, or neomycin due to the low frequency of resistant isolates. Similar models were specified for the following ES AMR outcomes: tetracycline, florfenicol, gamithromycin, tilmicosin, tildipirosin, tulathromycin, tylosin tartrate, and tiamulin.

Similar to the abovementioned limitations relevant to the rare outcomes (resistance in isolates), exposure to treatments may have been either non-identifiable with other factors or of low frequency, resulting in the inability to model such treatment effect. Due to the non-identifiability between DCT and herd effect, we were not able to estimate the distinct effect of DCT on AMR in fecal commensal bacteria. Specifically, only one herd reported the use of the procaine penicillin G and dihydrostreptomycin sulfate combination for DCT. Therefore, a comparison of the effect of DCT may have been confounded by the herd. In contrast, cephapirin sodium was used in our study herds (3 cows) for IMM treatment of clinical mastitis. However, we were not able to model the effect of its administration on AMR due to the low frequency of administration.

### Risk factors associated with antimicrobial resistance in *Escherichia coli*

Table 3 summarizes the final models for resistance in *E. coli* isolated from fecal samples of the study cows. The odds of resistance against ceftiofur, tetracycline, or trimethoprim-sulfamethoxazole in fecal *E. coli* were significantly higher in the winter than in the summer (*p* < 0.05). In addition, *E. coli* isolated from fecal samples from NCA study cows had lower odds of resistance against ceftiofur, tetracycline, sulfadimethoxine, or trimethoprim-sulfamethoxazole (*p* ≤ 0.05) in comparison to those from GSCA. Systemic administration of ceftiofur hydrochloride IM for treatment of lameness and unknown disease conditions during lactation was significantly associated with higher odds of tetracycline-resistant *E. coli* (*p* = 0.02). Cows with fecal scores of 2 had higher odds of shedding florfenicol-resistant *E. coli* (OR = 2.03) in comparison to cows with fecal scores of 1 (*p* = 0.02). In contrast, cows with fecal scores of 2 had lower odds of shedding trimethoprim-sulfamethoxazole-resistant *E. coli* (OR = 0.42) in comparison to cows with fecal score 1 (*p* < 0.01, Table 3).

**Table 3:** Final mixed-effects logistic regression model for risk factors associated with the prevalence of antimicrobial-resistant *E. coli* isolated from fecal samples collected on 10 California dairies from a random sample of 12 cows stratified by parity distribution on each of 10 California dairies and followed up from close up (approximately 2 weeks prior to calving) to 120 days in milk over two seasons (Winter and Summer).

Resistance against antimicrobial drugs (model outcome)	Predictor variable	Variable levels	Coefficient	Robust SE^1^	Odds ratio	OR 95% confidence interval		*p*-value
						Lower limit	Upper limit	
Ceftiofur	Region^2^	GSCA	Referent	–	–			
		NSJV	0.402	0.796	1.49	0.31	7.12	0.61
		NCA	−1.524	0.310	0.21	0.11	0.40	<0.01
	Sampling season	Winter	Referent	–	–			
		Summer	−1.704	0.695	0.18	0.04	0.71	0.01
	*Intercept*		−2.840	0.381	0.05	0.03	0.12	<0.01
	*Random effects*							
	Cow (intercept)		0.41	0.58		0.02	6.53	
Tetracycline	Region	GSCA	Referent	–	–			
		NSJV	−0.091	0.241	0.912	0.568	1.46	0.70
		NCA	−0.534	0.257	0.585	0.353	0.971	0.04
	Sampling season	Winter	Referent	–	–			
		Summer	−0.832	0.170	0.434	0.311	0.607	<0.01
	Ceftiofur hydrochloride (intramuscular)^3^	No	Referent	–				
		Yes	0.508	0.222	1.66	1.07	2.57	0.02
	sampling points, DIM	Close-up	Referent	–	–			
		30	−0.213	0.275	0.807	0.470	1.38	0.44
		60	−0.559	0.276	0.571	0.33	0.981	0.04
		90	−0.490	0.138	0.612	0.467	0.803	<0.01
		120	−0.466	0.210	0.627	0.415	0.947	0.03
	*Intercept*		−0.277	0.152	0.536	0.429	0.66	0.07
	*Random effects*							
	Cow (intercept)		0.128	0.064		0.048	0.341	
Florfenicol	Fecal score	1	Referent	–	–			
		2	0.709	0.30	2.032	1.116	3.700	0.02
		3	−0.390	0.76	0.676	0.152	3.008	0.61
	*Intercept*		2.682	0.19	14.62	9.935	21.53	<0.01
	*Random effects*							
	Cow (intercept)		0.55	0.27		0.21	1.44	
Sulfadimethoxine	Region	GSCA	Referent	–		–	–	
		NSJV	−0.168	0.281	0.844	0.486	1.467	0.55
		NCA	−0.635	0.314	0.529	0.285	0.981	0.04
	Sampling points, DIM	Close-up	Referent	–	–			
		30	−0.092	0.333	0.912	0.474	1.754	0.78
		60	−0.547	0.417	0.578	0.255	1.310	0.19
		90	−0.806	0.382	0.446	0.211	0.945	0.04
		120	−1.297	0.219	0.273	0.177	0.420	<0.01
	*Intercept*		0.546	0.278				0.05
	*Random effects*							
	Cow (intercept)		0.478	0.130		0.280	0.816	
Trimethoprim-sulfamethoxazole	Region	GSCA	Referent	–		–	–	
		NSJV	0.089	0.437	1.093	0.463	2.575	0.84
		NCA	−1.244	0.452	0.288	0.118	0.699	<0.01
	Sampling season	Winter	Referent	–	–			
		Summer	−2.165	0.457	0.114	0.046	0.281	<0.01
	Fecal score	1	Referent	–	–			
		2	−0.866	0.291	0.420	0.237	0.744	<0.01
		3	0.005	0.601	1.005	0.309	3.262	0.99
	*Intercept*		−1.415	0.319	0.242	0.129	0.453	<0.01
	*Random effects*							
	Cow (intercept)		<0.01	<0.01		<0.01	<0.01	

1 Standard error adjusted for clustering by study herds (*n* = 10).

2 GSCA, Greater Southern California; NSJV, Northern San Joaquin Valley; NCA, Northern California.

3 Ceftiofur hydrochloride for treatment of lameness and unknown disease condition.

### Risk factors associated with antimicrobial resistance in *Enterococcus*/*Streptococcus* spp. (ES)

Table 4 summarizes the final models for resistance in ES isolated from fecal samples of the study cows. The odds of resistance against tetracycline, florfenicol, tilmicosin, tildipirosin, or tiamulin in fecal ES were significantly higher in the summer than in the winter (*p* < 0.01). Fecal ES isolates from study cows in NCA had significantly lower odds of resistance against florfenicol, tilmicosin, tulathromycin, tildipirosin, or tiamulin than those from GSCA (*p* < 0.01). In addition, cows with fecal scores of 2 or 3 had higher odds of shedding florfenicol, tilmicosin, or tildipirosin-resistant ES in comparison to cows with fecal scores of 1 (*p* < 0.05), while only cows with fecal scores of 2 had higher odds of shedding tiamulin-resistant *E. coli* in comparison to cows with fecal scores of 1 (*p* = 0.04).

**Table 4:** Final mixed-effects logistic regression models for risk factors associated with the prevalence of antimicrobial-resistant *Enterococcus/Streptococcus* spp. isolated from fecal samples collected on 10 California dairies from a random sample of 12 cows stratified by parity distribution on each of 10 California dairies and followed up from close up (approximately 2 weeks prior to calving) to 120 days in milk over two seasons (Winter and Summer).

Resistance against antimicrobial drugs (model outcome)	Predictor variable	Variable levels	Co–efficient	Robust SE^1^	Odds ratio	95% Confidence interval		*p*–value
						Lower limit	Upper limit	
Tetracycline	Season	Winter	Referent	–		–	–	
		Summer	0.419	0.195	1.521	1.036	2.232	0.03
	*Intercept*		−1.696	0.272	0.183	0.107	0.312	<0.01
	*Random effects*							
	Cow (intercept)		0.827	0.313		0.393	1.739	
Florfenicol	Region^2^	GSCA	Referent	–	–			
		NSJV	−0.140	0.488	0.868	0.333	2.262	0.77
		NCA	−1.062	0.156	0.345	0.254	0.469	<0.01
	Season	Winter	Referent	–	–	–	–	
		Summer	0.743	0.151	2.103	1.561	2.832	<0.01
	Fecal score	1	Referent	–	–	–		
		2	0.322	0.146	1.349	1.014	1.793	0.03
		3	1.432	0.584	4.066	1.325	12.47	0.02
	*Intercept*		0.319	0.146	1.473	1.123	1.931	0.03
	*Random effects*							
	Cow (intercept)		0.087	0.145		0.003	2.28	
Tilmicosin	Region	GSCA	Referent	–	–			
		NSJV	0.069	0.572	1.071	0.348	3.291	0.90
		NCA	−0.865	0.321	0.421	0.224	0.790	<0.01
	Season	Winter	Referent	–		–	–	
		Summer	0.991	0.223	2.693	1.739	4.169	<0.01
	Fecal score	1	Referent	–	–			
		2	0.565	0.250	1.760	1.077	2.875	0.02
		3	1.266	0.476	3.549	1.396	9.022	<0.01
	*Intercept*		−0.014	0.307	0.985	0.539	1.798	0.96
	*Random effects*							
	Cow (intercept)		0.671	0.177		0.400	1.125	
Tulathromycin	Region	GSCA	Referent	–	–			
		NSJV	0.623	0.413	1.866	0.829	4.200	0.13
		NCA	−0.611	0.08	0.542	0.457	0.643	<0.01
	Sampling stage, DIM	Close–up	Referent					
		30	0.316	0.422	1.371	0.599	3.139	0.45
		60	0.733	0.224	2.081	1.341	3.229	<0.01
		90	0.251	0.598	1.285	0.397	4.157	0.67
		120	−0.321	0.642	0.725	0.206	2.549	0.62
	*Intercept*		−2.28	0.267	0.102	0.060	0.172	<0.01
	*Random effects*							
	Cow (intercept)		0.270	0.298		0.031	2.358	
Tildipirosin	Region	GSCA	Referent	–	–			
		NSJV	0.151	0.553	1.164	0.393	3.442	0.78
		NCA	−0.950	0.286	0.386	0.220	0.678	<0.01
	Season	Winter	Referent	–		–	–	
		Summer	1.062	0.258	2.892	1.742	4.801	<0.01
	Fecal score	1	Referent	–	–			
		2	0.537	0.261	1.712	1.025	2.858	0.04
		3	1.661	0.476	5.268	2.068	13.416	<0.01
	*Intercept*		0.052	0.296		0.588	1.885	0.86
	*Random effects*							
	Cow (intercept)		0.728	0.168	1.053	0.462	1.147	
Tylosin	Sampling stage, DIM	Close–up	Referent					
		30	1.347	0.592	3.848	1.204	12.291	0.02
		60	1.817	0.485	6.158	2.379	15.941	<0.01
		90	1.201	0.859	3.323	0.616	17.921	0.16
		120	0.627	0.919	1.872	0.310	11.34	0.49
	*Intercept*		−4.464	0.653	0.011	0.003	0.041	<0.01
	*Random effects*							
	Cow (intercept)		1.057	0.552		0.380	2.941	
Tiamulin	Region	GSCA	Referent	–	–			
		NSJV	0.197	0.615	1.218	0.364	4.074	0.75
		NCA	−1.313	0.287	0.268	0.152	0.472	<0.01
	Season	Winter	Referent	–		–	–	
		Summer	0.966	0.189	2.628	1.811	3.812	<0.01
	Sampling stage, DIM	Close–up	Referent					
		30	0.674	0.292	1.962	1.105	3.482	0.02
		60	0.643	0.360	1.903	0.938	3.859	0.07
		90	0.428	0.242	1.534	0.954	2.465	0.08
		120	0.731	0.327	2.079	1.093	3.951	0.03
	Fecal score	1	Referent	–	–			
		2	0.582	0.288	1.789	1.016	3.149	0.04
		3	1.369	0.732	3.935	0.936	16.530	0.06
	*Intercept*		−0.670	0.321	0.511	0.272	0.960	0.04
	*Random effects*							
	Cow (intercept)		1.104	0.294		0.655	1.861	

1 Standard error adjusted for clustering by study herds (*n* = 10).

2 GSCA, Greater Southern California; NSJV, Northern San Joaquin Valley; NCA, Northern California.

## Discussion

Our study demonstrated that AMD treatments and the location of the dairy and sampling season were significant risk factors associated with AMR in fecal commensal species in dairy cattle. However, AMR isolates were detected even in adult cows, even in the absence of exposure to specific drugs. BDCT was the most common indication for AMD use in the study’s dairy herds. Ceftiofur hydrochloride and cephapirin benzathine were the most frequent active substances used for BDCT, a finding that agrees with a prior survey of CA dairies (20). Similarly, a survey study conducted on Pennsylvania dairies (25) found that cephapirin benzathine was the most common DCT, followed by penicillins (procaine penicillin, cloxacillin) and third-generation cephalosporin (ceftiofur).

Cephalosporins and penicillin were the most common parenterally administered AMDs in the current study, in agreement with the Dairy 2014 study from the National Animal Health Monitoring System (NAHMS) (26), which reported that 73% of dairies nationwide used cephalosporins as the primary AMD, followed by penicillins, lincosamides, and tetracyclines. In our study, mastitis was the most commonly diagnosed indication for therapeutic use of AMD, followed by metritis and gastrointestinal disease conditions. Respondents to a previous survey of CA dairies also reported that clinical mastitis was the most common cause of AMD treatment (20). Our records showed that sulfadimethoxine was used for the treatment of clinical mastitis on one study dairy at a dose of 29 mg/kg/day for 5 days. However, there is no indication for using sulfadimethoxine to treat clinical mastitis (Sulfadimethoxine Injection 40%; Aspen Veterinary Resources, ^®^ Ltd.). Furthermore, sulfadimethoxine dosage per label calls for a loading dose on the first day of treatment (55 mg/kg/day), followed by half that dose for the remaining treatment days. In 2005, AVMA published a reminder on the prohibited extra-label drug use (ELDU) of sulfonamides in lactating dairy cattle and mentioned that unapproved use of sulfonamides is one of the most frequent causes of violative residues in food-producing animals (27). In addition, our records showed the use of ampicillin for the treatment of metritis and retained placenta. While ampicillin is an effective therapeutic AMD for the treatment of metritis, its use for the treatment of metritis in cattle is considered ELDU (28). Ceftiofur crystalline free acid (subcutaneous) was administered for treatment of clinical mastitis on a study dairy at the dose of 15 mL IM using a 2-dose regimen, which is considered ELDU because there is no label indication for clinical mastitis (EXCEDE^®^; Zoetis USA). Extra-label drug use must be approved by the prescribing veterinarian and used under the Animal Medicine Use Clarification Act (AMDUCA). Study herds’ treatment records indicated that 5.8% of cows treated with an AMD had no disease indication reported in the computer software. An unknown disease indication is possible if management recorded a treatment but not the indication. However, other possible reasons could be if the cows were treated for undiagnosed illness (e.g., fever of unknown origin) or other health findings that were not associated with a specific disease condition. Further outreach and extension education to veterinarians and dairy staff on following treatment protocols developed and updated regularly by the herd veterinarian may reduce the number of cows receiving AMD therapy designated under unknown illness.

### Risk factors associated with antimicrobial-resistant *Escherichia coli* isolated from fecal samples of dairy cattle

Our study reports on differences in resistance between the two seasons within the study period. Specifically, *E. coli* isolated from fecal samples in dairy farms during the study’s winter season were more resistant to ceftiofur, tetracycline, or trimethoprim-sulfamethoxazole than *E. coli* recovered during the summer. Possible reasons for the increased occurrence of AMR in *E. coli* during the winter season are the influence of temperature differences and AMD use. The winter season in CA can be associated with rain, which could provide suitable conditions for environmental bacteria to proliferate and increase the risk of clinical mastitis, metritis, and lameness, thus leading to increased antimicrobial usage. Seasonal differences in AMR were reported in similar studies. Massé et al. (29) found that extended-spectrum *β*-lactamase (ESBL)/AmpC-producing *E. coli* were recovered from fecal samples in dairy farms in Canada more frequently in the autumn season than in spring.

Although our study included 10 dairies across the three California milk sheds, it included 3 or 4 dairies in any one of the study regions, limiting the interpretation of differences in resistance between the study regions. The locations of the study dairies were a significant risk factor in the odds of AMR *E. coli* isolated from fecal samples of the study dairy cattle. Specifically, *E. coli* isolated from fecal samples of study cows in NCA had significantly lower odds of resistance against ceftiofur, tetracycline, sulfadimethoxine, or trimethoprim-sulfamethoxazole compared to those from GSCA and NSJV. The location differences reported in our study could be attributed to differences in management practices, environmental conditions, or herd size in the study herds. Amongst the management differences, two out of three study dairies in NCA did not report the use of any antimicrobials for the prevention and treatment of clinical mastitis in the study cows enrolled. On the remaining 8 study dairies, ceftiofur hydrochloride was the most common IMM AMD for treating clinical mastitis in lactating cows on six dairies, followed by cephapirin sodium on two dairies. Our finding is in agreement with Berge et al. (30), where the location of dairy farms was a significant factor in the odds of AMR *E. coli* isolated from cattle feces, attributing this difference to management-related factors. Regional differences in management practices within CA dairies were previously identified by (14), with dairies in NCA being significantly smaller in herd size, having fewer Holsteins compared to other milking cow breeds, and raising their own calves onsite compared to dairies in the GSCA and NSJV regions. The regional differences in AMR could also be attributed to the regional differences in climate and its associated management practices, such as exposure to pasture. Compared to southern regions, the NCA climate is milder and has more precipitation,^1^ allowing cows on one of the NCA dairies to be exclusively on pasture, on a second dairy with some pasture access, while the third had no access to pasture. In contrast, the higher temperatures and lower rainfall in NSJV and GSCA limited their dairy cow housing to dry lots or free stall pens. Regional differences in AMR prevalence were observed in previous studies (11). A Canadian study found that *Staphylococcus aureus* from cows in Ontario had significantly lower odds of tetracycline resistance compared to those from Québec (11). Another study conducted in Sweden (9) found that a higher prevalence of chloramphenicol resistance in *E. coli* was found in calves raised on farms in southern Sweden than in calves raised in northern Sweden.

In our study, systemic administration of ceftiofur hydrochloride for treatment of lameness and unknown disease was significantly associated with tetracycline resistance in *E. coli* isolates from fecal samples of the study dairy. Mann et al. (31) found that systemic antimicrobial treatment with ceftiofur hydrochloride decreased tetracycline susceptibility in fecal *E. coli* isolates in the treated group on day 2 in comparison to untreated cows and that isolates resistant to ceftiofur were more likely to show tetracycline resistance. At the gene level, Kanwar et al. (32) found an increase in the proportion of fecal *E. coli* isolates harboring *tet*(A) and *tet*(B) genes following ceftiofur treatment in feedlot cattle. Several studies on fecal *E. coli* have recorded that all ceftiofur-resistant isolates were found to be co-resistant to tetracycline because ceftiofur and tetracycline determinants are usually found together on the same plasmid (33, 34). Studies conducted on *Salmonella* isolates derived from cattle have indicated that the blaCMY-2 gene is usually located on a large IncA/C plasmid that harbors several other resistance genes, including tetracycline resistance genes (6, 34). Co-resistance to multiple classes of AMD, such as fluoroquinolones, tetracyclines, chloramphenicol, aminoglycosides, sulfonamides, and/or trimethoprim, has been reported in *E. coli* isolated from dairy cattle in the US (35).

Although ceftiofur was the most administered AMD in our study, *E. coli* isolates recovered from cows treated with ceftiofur were all susceptible to ceftiofur. Daniels et al. (36) reported that ceftiofur administration frequency did not impact levels of commensal *E. coli* containing the blaCMY-2 gene at the herd level. The absence of detection of the association between ceftiofur treatment and resistance in our study could be attributed to two possible factors. First, our fecal samples were collected monthly, which could have missed the resistance against ceftiofur in enteric *E. coli*, given that a recent study that compared resistance post-treatment at different ceftiofur concentrations showed loss of ceftiofur resistance at the 8 μg/mL level within 3–4 days post-treatment (8). Taylor et al. (37) found that on day 16 after cephalosporin treatment of cows with metritis, the population of cephalosporin-resistant bacteria in the treatment group was reduced by 0.5 log_10_ CFU. By day 28, the bacterial population returned to pre-treatment resistance levels. Second, the majority of ceftiofur used in our study dairies was IMM. Such non-parenteral administration may be less likely to confer resistance among fecal *E. coli* (37). In summary, changes in the AMD susceptibility of fecal *E. coli* were associated with the choice of AMD, geographical location, and sampling season, which suggests that AMR is a multifactorial phenomenon.

### Risk factors associated with antimicrobial-resistant *Enterococcus/Streptococcus* spp.

Cow-level ES AMR incidence ranged from 1.7% for penicillin to >90% for florfenicol and macrolides (tildipirosin or tilmicosin), which showed that enteric ES had a higher rate of AMR for drugs not approved for use in lactating dairy cows despite the study herds’ treatment records showing no administrations of macrolides or florfenicol in adult cows. Other studies similarly reported AMR to florfenicol and macrolides in *Enterococcus* spp. isolated from dairy cows (38, 39). Another study reported that the predominant AMR genes in *Enterococcus* spp. from dairy cattle in CA targeted macrolide drugs (40). The high levels of resistance for florfenicol and macrolides in enteric ES detected in our study could be attributed to the mechanism of co-selection, similar to that related to systemic administration of ceftiofur hydrochloride and its associated increase in tetracycline resistance in *E. coli*. Florfenicol resistance in *E. coli* is associated with the floR gene, which is co-located on plasmids with genes that confer resistance to other antimicrobial drugs, which may explain florfenicol resistance spread with exposure to drugs other than florfenicol (40). In our study herds, the common practice of flushing free-stall pens with recycled lagoon water may have played a role in exposing adult cattle to resistant bacteria or AMD residues in excreta or secretions of cows and/or calves treated with AMD. Florfenicol and macrolides are commonly used to treat respiratory diseases in calves. Hence, recycled lagoon water may have exposed adult cows to the florfenicol and macrolide residues from treated calves.

Regional differences in cows harboring resistant ES were also observed in our study, with lower odds of having cows harboring ES resistance to florfenicol, tilmicosin, tulathromycin, tildipirosin, or tiamulin-resistant ES in NCA in comparison to cows in GSCA and NSJV regions. Previous studies reported that different bacteria with varying AMR exist in different regions (13, 41, 42). Farms have unique ecosystems composed of each farm’s environment, geography, weather, and management factors that may serve as suitable media for amplifying and disseminating AMR in bacteria (43). Another study reported regional differences in AMR prevalence in Canada. It attributed those findings to differences in management, environment, geography, weather, and resource availability that might influence AMR. However, differences were only observed for bovine mastitis *Staphylococcus aureus* but not gram-negative pathogens (11, 12).

The presence of AMR in adult cows, despite the absence of specific drug exposure, may be linked to environmental contamination with resistant bacteria, direct colonization, or horizontal gene transfer. In addition, drug residues from treated calves may expose adult cows through the recycled flush system. Further research is required to confirm such hypotheses. The current study identified a high proportion of florfenicol resistance in *E. coli* and ES on the study dairies with no record of florfenicol use in the adult herd. This drug is not currently labeled for use in dairy cattle 20 months or older. Therefore, the link between AMD treatment of youngstock and AMR of fecal commensal bacteria in adult cows in the same herds needs further investigation.

## Limitations

In addition to the residual confounding characteristic of our observational study design, the current study herds were a convenience sample of CA dairies and not a random sample of the state’s dairies. Experimental study designs, such as double-blind, complete block, randomized trials, would be more suitable than observational studies to investigate the causal association between exposure to a single drug and antimicrobial resistance against it without accounting for sources of variability in the real world. The use of selective media for the isolation of *Enterococcus* spp. selected both *Enterococcus* spp. and *Streptococcus* spp., only recently distinguished, could have introduced a bias (13). While the spatial distribution of the herds captured the state’s three milk sheds identified in earlier studies, it is possible that the study herds were not representative. Our study was conducted on dairy cows from close-up (2 weeks pre-calving) to 120 DIM only. It could not provide information on the epidemiology of AMD use and resistance later in the lactation.

The current estimates of the DDDstudy may not accurately reflect the actual amount of drug administered per kg per day on a study dairy if the body weight of a treated cow differs from the assumed weight of 680 kg used for calculating the DDDstudy. Actual administered DDDstudy estimates would have required knowledge of each cow’s body weight, data that could have been but was not collected.

The observed associations were based on AMD use reported by dairies using computer management systems or paper records and not based on empty vial verification. However, dairy owners allowed us full access to the study herds and their dairy records during the study period. Further, the study associations were modeled using the phenotypic data with no information on determinants of resistance.

Several drugs and their resistance patterns were not modeled due to their infrequent use or observation. Several drugs and their resistance patterns could not be modeled due to their low frequency of use or observation, respectively.

Specifically, each of the eight dairies that implemented dry cow AMD therapy administered only a single AMD, confounding any comparison by herd-specific factors. In addition, only one dairy used a combination of procaine penicillin G and dihydrostreptomycin sulfate, leading to perfect non-identifiability due to the lack of variability in treatment across the study sample. A more robust experimental approach, such as comparing dry cow IMM AMD treatment with untreated controls and more frequent sampling of milk post-calving, would provide a clearer assessment of this association. A recent study of a random sample of non-aureus *Staphylococcus* spp. isolated from milk samples collected at dry-off and post-calving found increased resistance to cephalothin, ceftiofur, and oxacillin in cows treated at dry-off with AMD of the same class or a class with a shared resistance mechanism (44).

## Conclusion

Our study showed that florfenicol, tetracycline, or sulfadimethoxine-resistant *E. coli* and florfenicol, tildipirosin, tilmicosin, or tiamulin-resistant ES were commonly isolated from feces of the study dairy cows in CA. Extra-label use of AMD was observed in sporadic cases in our study dairies. The finding of AMD-resistant *E. coli* and ES isolates from the feces of dairy cows was associated with multiple factors. Systemic administration of ceftiofur hydrochloride for treatment of lameness and unknown disease during lactation was significantly associated with higher odds of isolating fecal *E. coli* resistant to tetracycline. Regional and seasonal differences were reported in our study, which could be attributed to differences in management practices, environmental conditions, or herd size in California dairies. Resistance to calf hood AMD in adult dairy cattle was observed. Therefore, follow-up research is needed to understand the association between the use of AMD on a dairy, including for calf treatments, and AMR.

## Data Availability

The datasets presented in this article are not readily available because the study data were collected under Food and Agriculture Law which requires maintaining confidentiality of the participating farms including publishing only aggregate analyzed data. This study was sponsored by the California Department of Food and Agriculture and is subject to California Food and Agriculture Code (FAC) Sections 14400 to 14408. FAC section 14407 requires that data collected be held confidential to prevent individual identification of a farm or business. Reasonable requests for additional data in aggregate form can be provided by contacting Dr. Sharif Aly, email: SAly@ucdavis.edu.

## Footnotes

^1^ https://wrcc.dri.edu/Climate/narrative_ca.php
