# Supplementary material for: Antimicrobial drug use and its association with antimicrobial resistance in fecal commensals from cows on California dairies
Source: Front Vet Sci. 2025 Feb 10;11:1504640. doi: 10.3389/fvets.2024.1504640 (PMC11848851; doi:10.3389/fvets.2024.1504640)
Supplement: Supplementary file 1 [file Table_1.docx]

**Supplementary materials:**

**Antimicrobial drug use and its association with antimicrobial resistance in fecal commensals from cows on California dairies**

Essam M. Abdelfattah^1,2^, Pius Ekong^1^, Emmanuel Okello^1,3^, Tapakorn Chamchoy^1^, Betsy M. Karle^4^, Randi Black^5^, Wagdy ElAshmawy^1,6^, David Sheedy^1^, Deniece R. Williams^1^, Terry W. Lehenbauer^1,3^, Barbara A. Byrne^7^ and Sharif S. Aly^1,3*^

^1^Veterinary Medicine Teaching and Research Center, School of Veterinary Medicine, University of California Davis, Tulare, CA

^2^Department of Animal Hygiene, and Veterinary Management, Faculty of Veterinary Medicine, Benha University, Qalubiya Governorate 13511, Egypt

^3^Department of Population Health & Reproduction, School of Veterinary Medicine, University of California Davis, CA

^4^Cooperative Extension, Division of Agriculture and Natural Resources, University of California, Orland, CA

^5^Cooperative Extension, Division of Agriculture and Natural Resources, University of California, Santa Rosa, CA

^6^Department of Internal Medicine and Infectious Diseases, Faculty of Veterinary Medicine, Cairo University, Giza, Egypt.

^7^Department of Pathology, Microbiology, and Immunology, School of Veterinary Medicine, University of California Davis, CA

* Corresponding author: Dr. Sharif Aly.18830 Rd 112, Tulare, CA, 93277, USA; Email address: [saly@ucdavis.edu](mailto:saly@ucdavis.edu)

**Table S1.** Herd level descriptive statistics across 10 California dairies for anti-microbial drugs (AMD) administered to a random sample of 12 cows stratified by parity on each of 10 premises and followed up from close-up (approximately two weeks prior to calving) to 120 days in milk over two seasons (Winter and Summer) for a total of 240 cows.

| Region | Herd | Active substance | Route | Days per regimen | Number of cows^1^ |
| --- | --- | --- | --- | --- | --- |
| Greater Southern CA | 1 | Ceftiofur hydrochloride | IMM^2^  (Dry cow therapy) | 1 | 16 |
|  |  | Ceftiofur hydrochloride | IM^3^ | 3-5 | 3 |
|  |  | Ceftiofur hydrochloride | IMM | 8 | 5 |
|  |  | Ceftiofur hydrochloride | SQ^4^ | 3 | 3 |
|  |  | Pirlimycin hydrochloride | IMM | 3 | 1 |
|  | 2 | Ceftiofur hydrochloride | IMM  (Dry cow therapy) | 1 | 20 |
|  |  | Ceftiofur hydrochloride | IMM | 4 | 3 |
|  | 3 | Cephapirin benzathine | IMM  (Dry cow therapy) | 1 | 18 |
|  |  | Ceftiofur hydrochloride | IMM | 1-2 | 1 |
|  |  | Ceftiofur hydrochloride | IM | 5 | 1 |
|  | 4 | Cephapirin benzathine | IMM  (Dry cow therapy) | 1 | 18 |
|  |  | Ceftiofur hydrochloride | IMM | 3 | 5 |
|  |  | Cephapirin sodium | IMM | 1 | 1 |
|  | 5 | Ceftiofur hydrochloride | IMM  (Dry cow therapy) | 1 | 20 |
|  |  | Ceftiofur hydrochloride | IM | 3-5 | 10 |
|  |  | Ceftiofur hydrochloride | IMM | 5 | 1 |
| Northern San Joaquin Valley | 6 | Ceftiofur hydrochloride | IMM  (Dry cow therapy) | 1 | 19 |
|  |  | Ceftiofur hydrochloride | IMM | 3 | 2 |
|  |  | Ceftiofur crystalline free acid | SQ | 2 | 2 |
|  |  | Pirlimycin hydrochloride | IMM | 4 | 2 |
|  |  | Sulfadimethoxine | IV | 5 | 1^6^ |
|  | 7 | Cephapirin benzathine | IMM  (Dry cow therapy) | 1 | 20 |
|  |  | Cephapirin sodium | IMM | 1 | 3 |
|  |  | Ampicillin | IM | 3 | 2 |
| Northern CA | 8 | Procaine Penicillin G - dihydrostreptomycin sulfate combination | IMM  (Dry cow therapy) | 1 | 13 |
|  | 9 | No AMD treatments reported in the 24 study cows during study period | - | - | - |
|  | 10 | No AMD treatments reported in the 24 study cows during study period | - | - | - |

^1^Cows within the same herd may have more than one regimen of the same or different AMD; ^2^IMM: intramammary;
^3^IM: intramuscular;
^4^SQ: subcutaneous.

**Table S2.** Grams per regimen (g/reg) for each active substance administered across 10 California dairies to a random sample of 12 cows stratified by parity distribution on each of 10 premises and followed up from close-up (approximately two weeks prior to calving) to 120 days in milk over two seasons (Winter and Summer) for a total of 240 cows.

| Use category (Number of cows) | Active substance  (Number of regimens) | Route | Mean  g/reg | Median  g/reg | SD | Minimum  g/reg | Maximum  g/reg |
| --- | --- | --- | --- | --- | --- | --- | --- |
| Dry cow^1^ (144) | Ceftiofur hydrochloride (75) | IMM | 2 | 2 | 0 | 2 | 2 |
|  | Cephapirin benzathine (56) | IMM | 1.2 | 1.2 | 0 | 1.2 | 1.2 |
|  | Procaine Penicillin G/ dihydrostreptomycin sulfate combination (13) | IMM | 3.38/ 3.38 | 4.00/ 4.00 | 0.96/ 0.96 | 2.00/ 2.00 | 4.00/ 4.00 |
| Clinical mastitis (21) | Ceftiofur crystalline free acid (1) | SC | 6 | 6 | - | 6 | 6 |
|  | Ceftiofur hydrochloride (21) | IMM | 0.57^2^ | 0.5 | 0.29 | 0.25 | 1 |
|  | Cephapirin sodium (7) | IMM | 0.37 | 0.4 | 0.08 | 0.2 | 0.4 |
|  | Pirlimycin hydrochloride (4) | IMM | 0.19 | 0.2 | 0.025 | 0.15 | 0.2 |
|  | Sulfadimethoxine (1) | IV | 100 | 100 | - | 100 | 100 |
| Lameness (1) | Ceftiofur hydrochloride (1) | IMM | 5 | 5 | - | 5 | 5 |
| Metritis (3) | Ampicillin (2) | IMM | 18.75 | 18.75 | 0 | 18.75 | 18.75 |
|  | Ceftiofur hydrochloride (1) | SC | 2.25 | 2.25 | - | 2.25 | 2.25 |
| Gastro- intestinal (2) | Ceftiofur hydrochloride (2) | SC | 2.63 | 2.625 | 0.53 | 2.25 | 3 |
| Unknown^3^ (14) | Ceftiofur crystalline free acid (1) | SC | 6 | 6 | - | 6 | 6 |
|  | Ceftiofur hydrochloride (13) | IM | 3.29 | 3.75 | 0.58 | 2.25 | 3.75 |

^1^Two of the 10 study dairies did not administer dry cow therapy at dry-off and their enrolled study cows received no AMD during the study period.
^2^Of the cows with clinical mastitis cases, one cow was treated for severe clinical mastitis using ceftiofur hydrochloride administered intramammary (label dose 0.125 g/admin) at diagnosis and repeated in 12 hours (1 day regimen). The two administrations affect only the grams per regimen and not grams per administration (Supplement 1, lactating cows AMD, cow index 49).
^3^Cow received antibiotic treatment without a reported disease condition or specified cause.

**Table S3.** Grams per administration (g/admin) for each active substance administered across 10 California dairies to a random sample of 12 cows stratified by parity distribution on each of 10 premises and followed up from close up (ap-proximately two weeks prior to calving) to 120 days in milk over two seasons (Winter and Summer) for a total of 240 cows.

| Use category (Number of cows) | Active substance (Number of administrations) | Route | Mean  g/admin | Median g/admin | SD | Minimum g/admin | Maximum g/admin |
| --- | --- | --- | --- | --- | --- | --- | --- |
| Dry cow^1^  (144) | Ceftiofur hydrochloride (75) | IMM | 2.00 | 2.00 | 0.00 | 2.00 | 2.00 |
|  | Cephapirin benzathine (56) | IMM | 1.20 | 1.20 | 0.00 | 1.20 | 1.20 |
|  | Procaine Penicillin G/ dihydrostreptomycin sulfate combination (13) | IMM | 3.38/ 3.38 | 4.00/ 4.00 | 0.96/ 0.96 | 2.00/ 2.00 | 4.00/ 4.00 |
| Clinical mastitis (21) | Ceftiofur crystalline free acid (1) | SC | 3.00 | 3.00 | - | 3.00 | 3.00 |
|  | Ceftiofur hydrochloride (96) | IMM | 0.125^2^ | 0.125 | 0 | 0.125 | 0.125 |
|  | Cephapirin sodium (13) | IMM | 0.20 | 0.20 | 0 | 0.20 | 0.20 |
|  | Pirlimycin hydrochloride (15) | IMM | 0.05 | 0.05 | 0 | 0.05 | 0.05 |
|  | Sulfadimethoxine (5) | IV | 20.00 | 20.00 | - | 20.00 | 20.00 |
| Lameness (1) | Ceftiofur hydrochloride (5) | IM | 1.00 | 1.00 | - | 1.00 | 1.00 |
| Metritis  (3) | Ampicillin (6) | IM | 6.25 | 6.25 | 0.00 | 6.25 | 6.25 |
|  | Ceftiofur hydrochloride (3) | SC | 0.75 | 0.75 | - | 0.75 | 0.75 |
| Gastro-intestinal (2) | Ceftiofur hydrochloride (6) | SC | 0.88 | 0.88 | 0.18 | 0.75 | 1.00 |
| Unknown^3^  (14) | Ceftiofur crystalline free acid (2) | SC | 3.00 | 3.00 | - | 3.00 | 3.00 |
|  | Ceftiofur hydrochloride (57) | IM | 0.75 | 0.75 | 0.00 | 0.75 | 0.75 |

^1^ Two of the 10 study dairies did not administer dry cow therapy at dry-off and their enrolled study cows received no AMD during the study period.
^2^Of the cows with clinical mastitis two had multiple quarters affected simultaneously. The first cow had two quarters affected simultaneously and treated with ceftiofur hydrochloride administered intramammary; the second had all 4 quarters affected simultaneously and treated with pirlimycin hydrochloride administered intramammary. Such multiple quarters affected with clinical mastitis does not change the dose at the quarter level, yet it is imperative to adjust the grams per administration to adjust for the multiple quarters affected simultaneously using a coefficient that represents the number of quarters affected.
^3^Cow received antibiotic treatment without specified cause entered in records.

**Table S4.** Administration per regimen for each active substance administered across 10 California dairies to a random sample of 12 cows stratified by on each of 10 premises and followed up from close-up (approximately two weeks prior to calving) to 120 days in milk over two seasons (Winter and Summer) for a total of 240 cows.

| Use category  (Number of cows) | Active substance | Route | Mean  Admin^1^ | Median  Admin | SD | Minimum  Admin | Maximum  Admin |
| --- | --- | --- | --- | --- | --- | --- | --- |
| Dry cow^2^ (144) | Ceftiofur hydrochloride | IMM | 1 | 1 | 0 | 1 | 1 |
|  | Cephapirin benzathine | IMM | 1 | 1 | 0 | 1 | 1 |
|  | Procaine Penicillin G/ dihydro-streptomycin sulfate combination | IMM | 1 | 1 | 0 | 1 | 1 |
| Clinical mastitis (21) | Ceftiofur crystalline free acid | SC | 2 | 2 | - | 2 | 2 |
|  | Ceftiofur hydrochloride | IMM | 4.57 | 4 | 2.34 | 2 | 8 |
|  | Cephapirin sodium | IMM | 1.86 | 2 | 0.38 | 1 | 2 |
|  | Pirlimycin hydrochloride | IMM | 3.75 | 4 | 0.5 | 3 | 4 |
|  | Sulfadimethoxine | IV | 5 | 5 | - | 5 | 5 |
| Lameness (1) | Ceftiofur hydrochloride | IM | 5 | 5 | - | 5 | 5 |
| Metritis  (3) | Ampicillin | IM | 3 | 3 | 0 | 3 | 3 |
|  | Ceftiofur hydrochloride | SC | 3 | 3 | - | 3 | 3 |
| Gastro- intestinal (2) | Ceftiofur hydrochloride | SC | 3 | 3 | 0 | 3 | 3 |
| Unknown^3^  (14) | Ceftiofur crystalline free acid | SC | 2 | 2 | - | 2 | 2 |
|  | Ceftiofur hydrochloride | IM | 4.38 | 5 | 0.77 | 3 | 5 |

^1^Admin, administration;
^2^Two of the 10 study dairies did not administer dry cow therapy at dry-off and their enrolled study cows received no AMD during the study period.
^3^Cow received antibiotic treatment without specified cause entered in records.
